# A biologically informed method for detecting rare variant associations

**DOI:** 10.1186/s13040-016-0107-3

**Published:** 2016-08-30

**Authors:** Carrie Colleen Buchanan Moore, Anna Okula Basile, John Robert Wallace, Alex Thomas Frase, Marylyn DeRiggi Ritchie

**Affiliations:** 1 Duke University Medical Center, Duke General Surgery, Durham, NC 27710 USA; 2 Department of Biochemistry and Molecular Biology, Center for Systems Genomics, The Pennsylvania State University, University Park, PA 16802 USA; 3 Biomedical and Translational Informatics, Geisinger Health System, Danville, PA 17821 USA

## Abstract

**Background:**

BioBin is a bioinformatics software package developed to automate the process of binning rare variants into groups for statistical association analysis using a biological knowledge-driven framework. BioBin collapses variants into biological features such as genes, pathways, evolutionary conserved regions (ECRs), protein families, regulatory regions, and others based on user-designated parameters. BioBin provides the infrastructure to create complex and interesting hypotheses in an automated fashion thereby circumventing the necessity for advanced and time consuming scripting.

**Purpose of the study:**

In this manuscript, we describe the software package for BioBin, along with type I error and power simulations to demonstrate the strengths and various customizable features and analysis options of this variant binning tool.

**Results:**

Simulation testing highlights the utility of BioBin as a fast, comprehensive and expandable tool for the biologically-inspired binning and analysis of low-frequency variants in sequence data.

**Conclusions and potential implications:**

The BioBin software package has the capability to transform and streamline the analysis pipelines for researchers analyzing rare variants. This automated bioinformatics tool minimizes the manual effort of creating genomic regions for binning such that time can be spent on the much more interesting task of statistical analyses. This software package is open source and freely available from http://ritchielab.com/software/biobin-download

**Electronic supplementary material:**

The online version of this article (doi:10.1186/s13040-016-0107-3) contains supplementary material, which is available to authorized users.

## Background

Recent advances in sequencing technology and drastic decreases in cost have facilitated the generation of a prolific amount of sequence data. This has presented an opportunity for the investigation of low frequency and rare sequence variants beyond traditional genome-wide association (GWA) based approaches. Rare variants have recently been implicated in multifactorial conditions ranging from neurodegenerative diseases like Alzheimer’s and Parkinson’s disease, to metabolic disorders, such as obesity, and various cancers, including both prostate and lung cancer [1–6]. Elucidating the influence of rare variants on common diseases may expand our understanding of the heritability of complex traits, and it may eventually provide information that is useful to clinical patient care through the implementation of personalized, preventive practices.

Even with increased data availability, progress toward understanding rare genomic variation and its association to common human disease lags behind technological sequencing advances. Scientists are hindered in exploiting these advances because strategies for analyzing these data are underdeveloped. The growing disparity in rapidly advancing data collection versus slowly developing data analysis methods mandates a more concerted research effort to develop the necessary analytical tools for successful interpretation of genetic and biological data. Tools designed specifically for rare and low-frequency variant analysis require special considerations as these variants are individually uncommon, and often statistically underpowered for detecting phenotypic association [7, 8]. Also, the large sample size requirements may be prohibitive [9]. To increase the composite allele frequency and analyze smaller sample sizes, collapsing or binning methods are commonly utilized. Collapsing methods aggregate variants into a single genetic variable, which can then be used for subsequent statistical analysis, thereby reducing the number of degrees of freedom and also improving power in the analysis.

Many previous strategies developed for rare variants have focused on the statistical analysis of a pre-defined region rather than how to best group variants in an informative manner. Agnostic or un-informed binning approaches can often lead to a decrease in power when there are variants with different directions of effect or too many neutral variants that mitigate the signal. The most successful collapsing method groups variants likely to have an impact on the function of a specific gene or genomic unit and compares the variant distribution or composite genetic score distribution across the trait of interest.

BioBin [10–12] is a novel bioinformatics tool developed for the multi-level binning of rare variants using a biological knowledge-driven framework. BioBin collapses variants into user-designated biological features such as genes, pathways, evolutionary conserved regions (ECRs), protein families, regulatory regions, and others. Further, BioBin provides the infrastructure to create complex and interesting hypotheses in an automated fashion thereby circumventing the necessity for advanced and time consuming scripting. Simulation testing highlights the utility of BioBin as a fast, comprehensive and expandable tool for the biological binning and analysis of low-frequency variants in sequence data. While multiple biological applications of BioBin have previously been described [10–13], the manuscript herein concentrates on the software features, specifications and various analysis options within the BioBin package. We focus on presenting a comprehensive description of the capabilities of BioBin to provide a resource for users to tailor binning analyses to their specific hypotheses. Additionally, we demonstrate the utility of this software through type I error and power simulations. The BioBin software package has the capability to transform and streamline analysis pipelines for researchers analyzing rare variants in DNA sequencing data. This automated bioinformatics tool minimizes the manual task of curating biologically-relevant regions for binning, such that efforts can instead be spent on subsequent statistical analyses. This software package is open source and freely available from http://ritchielab.com/software/biobin-download.

## Implementation

**Fig. 1:**
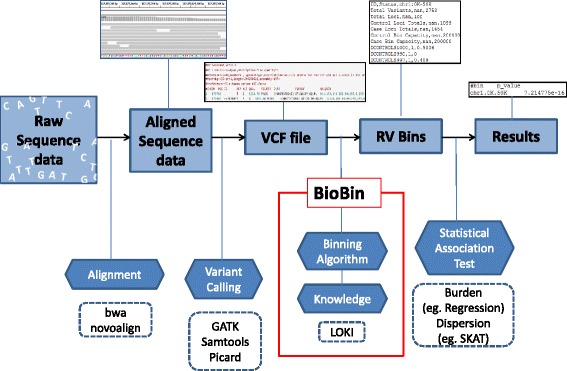
Rare variant analysis pipeline. General rare variant analysis pipeline starting with raw sequence data and ending with association analysis results. BioBin accepts VCF files and is able to bin variation in biologically informed boundaries using an internal biological knowledge biorepository, LOKI. BioBin’s output easily facilitates use of various statistical tests

BioBin is a unified command line bioinformatics tool for the biologically-inspired binning of rare variants. The novelty of BioBin is the automated multi-level binning process, rather than a focus on a particular statistical test. BioBin frees users from the tedious task of manually curating biologically important regions from multiple sources by using information from publicly available resources. The role of BioBin in a typical rare variant analysis pipeline is illustrated in Fig. 1. BioBin accepts VCF files and utilizes an internal binning algorithm in conjunction with biological information from an internal repository known as LOKI (described in a subsequent section). The algorithm will bin sequence variation in user-selected biologically-defined boundaries. The user then has the freedom to choose a specific statistical test for association.

### BioBin resource requirements

BioBin is a stand-alone command line application written in C++ that relies on a locally built Library of Knowledge Integration (LOKI) database to create knowledge-based bins. Source distributions are available for Mac and Linux operating systems and require minimal prerequisites to compile. The BioBin distribution includes tools that allow the user to create and update the LOKI database by downloading information directly from source websites. BioBin is open-source and publicly available for download on the Ritchie lab website (https://ritchielab.com/software/biobin-download).

**Fig. 2:**
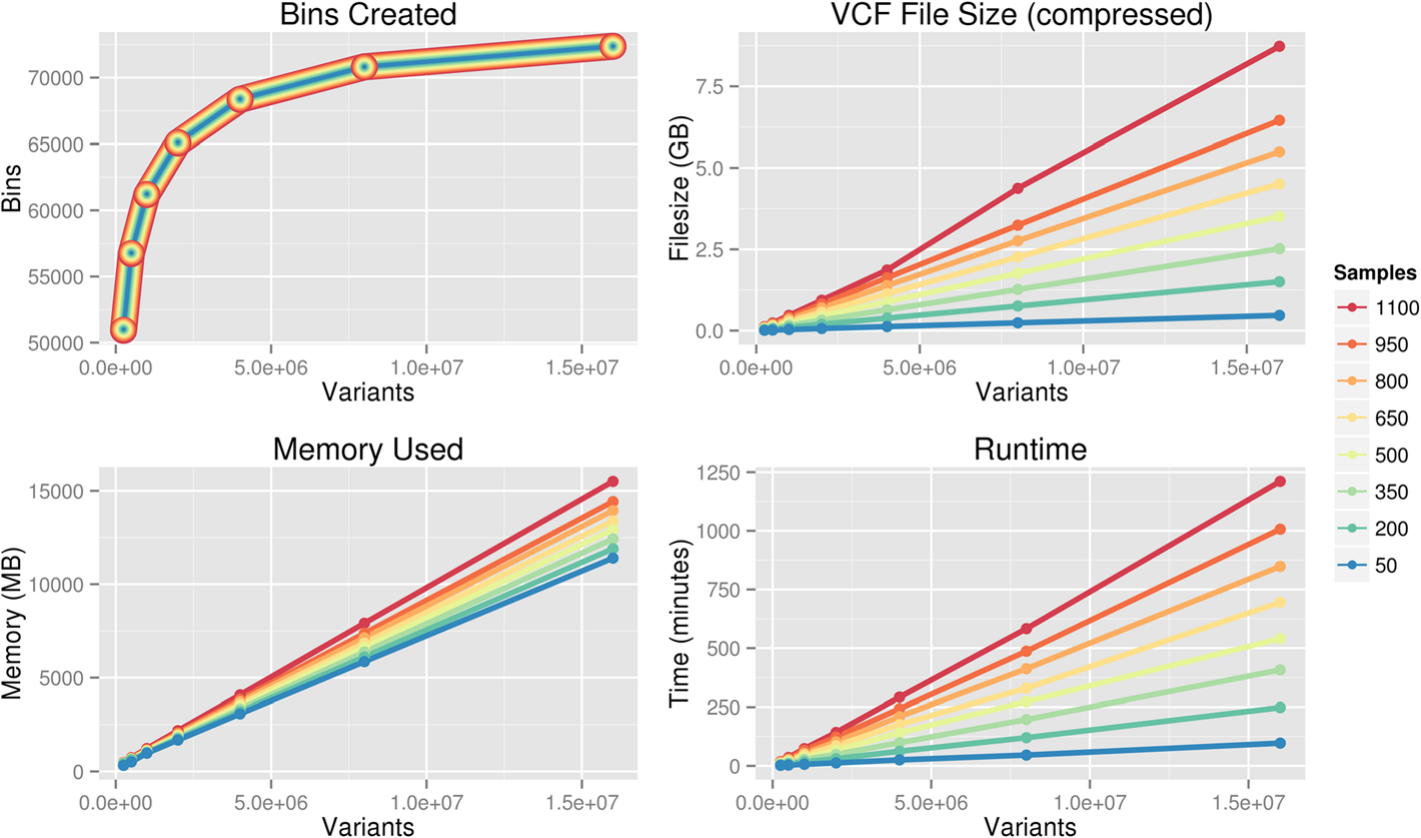
BioBin resource requirements. BioBin resource requirements for varying numbers of study variants and sample sizes. The number of variants is the primary driver of needed resources, with the number of variants increasing the runtime due to the size of the input file

To evaluate the computational requirements of BioBin, we randomly selected a number of variants and a number of individuals from the 1000 Genomes Project Phase I low coverage data [14] and applied a BioBin gene binning analysis to the resulting dataset. Because the minor allele frequency dramatically impacts the selection of variants to be binned, we set parameters to include all variants, regardless of rarity, to produce consistent results. Over 10 replicates, Fig. 2 shows that bin generation is highly correlated to the number of loci (or genomic positions) in the study and both the number of loci and bin generation drive the memory requirements. The number of individuals in a study does not have a large impact on resource requirements, but does increase the size of the input VCF file and thus the time it takes BioBin to read the input VCF file. Even with large datasets, BioBin can be run relatively quickly without access to specialized computer hardware or a computing cluster; however, the number of low frequency variants to be binned is the primary driver of memory usage. Running a gene-based analysis of targeted exome capture of 82 pharmacogenes for 8194 samples [15], BioBin took approximately 10 min and 150 MB of RAM using a single core of an Intel Xeon E5-2670 processor. Using linear extrapolation based on the size of the target, we expect that a gene-based whole exome analysis of a similarly sized population would take approximately 6 h and 6GB of memory.

### BioBin software features

#### Library of Knowledge Integration (LOKI)

**Fig. 3:**
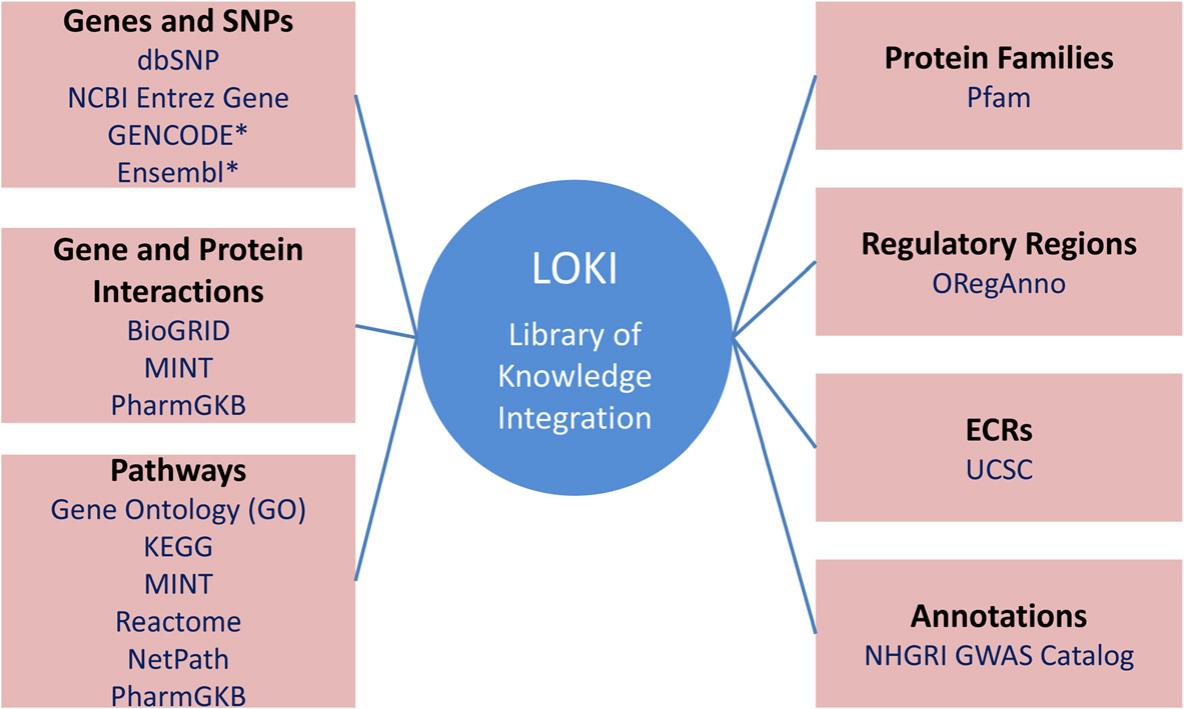
LOKI. BioBin collapses variants into biological features by consulting LOKI, an internal biorepository that integrates multiple resources from the public domain. All databases within LOKI are listed below their corresponding feature category. Databases annotated with an asterisks (*) represent sources that will be present in the version 3 release of LOKI

BioBin relies on the Library of Knowledge Integration (LOKI), which integrates multiple databases providing a comprehensive biological knowledge platform for variant binning [16]. LOKI is a database that contains biological information from resources including the National Center for Biotechnology (NCBI) dbSNP and gene Entrez [17], Kyoto Encyclopedia of Genes and Genomes (KEGG) [18], Reactome [19], Gene Ontology (GO) [20], Protein families database (Pfam) [21], NetPath- signal transduction pathways [22], and others. Figure 3 provides a complete list of databases within LOKI. LOKI provides a standardized interface and terminology to disparate sources, each containing individual means of representing data [16, 23]. The four main concepts used in LOKI are *position*, *region*, *group*, and *source. Position* refers to the chromosome and base-pair position of single variants, such as single nucleotide variants (SNVs). A *region* represents any genomic segment with a start and stop position including genes, copy number variants (CNVs), insertions or deletions, and evolutionary conserved regions (ECRs). *Sources* are the external databases compiled in LOKI that contain *groups* of interconnected information, thus organizing the data in a standardized manner. For example, BioGrid ID:468346 defines a *group* from the BioGrid data *source* which contains the following *regions*: HMGB1P1, CTCFL, and PRMT7.

LOKI is implemented in SQLite, a relational database management system, which does not require a dedicated database server. A system initially building LOKI should have approximately 100GB of disk storage available for the LOKI database file, the LOKI source data, and space for python installer scripts. An updater script will automatically process and combine information from the various sources into a single database file (some of the temporary files are removed during this process). Once the build is complete, the LOKI database file required to run BioBin will be under 25GB. The script to build LOKI is open source, publicly available on the Ritchie lab website, and is included with the BioBin software. Users with knowledge of relational databases can customize their LOKI database by including or excluding sources, providing additional sources, and updating source information as frequently as needed [16].

#### Multi-level binning and filtering

**Fig. 4:**
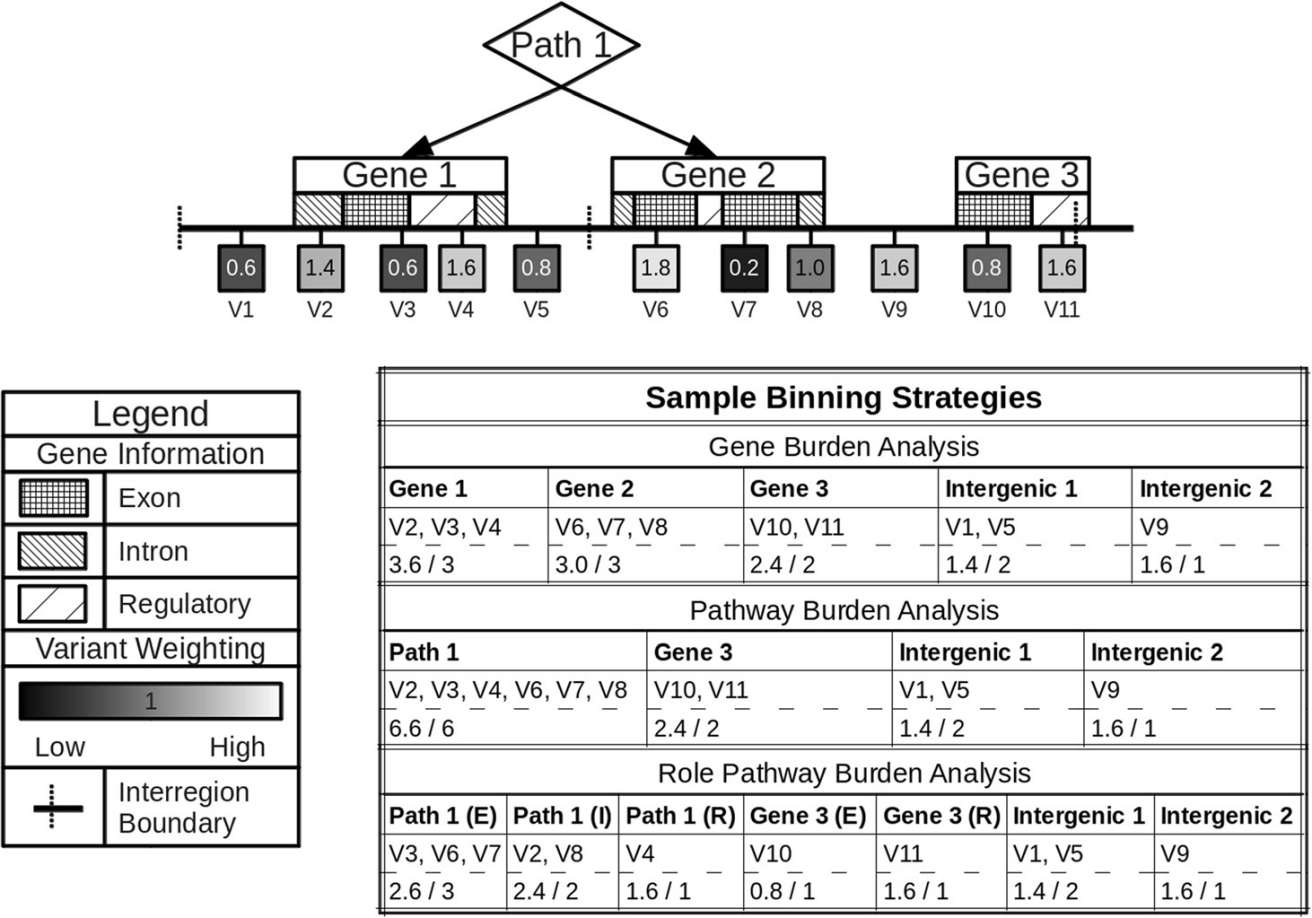
Binning strategies in BioBin. Alternate binning strategies using biological knowledge (gene information) and functional or role annotations (variant information). Three example binning strategies are shown: gene burden analysis, pathway burden analysis, and functional pathway burden analysis. Note the intergenic bins that collects variants fall outside of the binning strategy

The novelty of BioBin is its ability to automate bin generation at multiple levels of biological knowledge into one streamlined analysis. Figure 4 provides example binning strategies using biological information in LOKI. Using hierarchical biological relationships and optional functional or role information, BioBin can create many variant combinations to bin. As a standard in the current iteration of LOKI, NCBI dbSNP and NCBI Entrez Gene have been selected as the primary sources of position and regional information due to the data quality, reliability, and clearly defined database schema. These sources also most closely correspond to the region and group IDs provided by other database sources integrated into LOKI.

In addition to binning variants based on knowledge, BioBin also provides an option to bin variants that do not associate with any available knowledge. These are known as inter-region bins, or if generated between gene features, intergenic bins. After feature selection using LOKI and/or external custom files, inter-region bins can be created using a configurable width parameter (in kb). These bins catch variants that do not fit into biologically defined feature types (see intergenic bin labels in Fig. 4). For example, if one were testing low frequency burden differences between two groups across genes, all variants in genes would be collapsed into respective gene bins, and variants outside of gene boundaries would be binned based on genomic location in intergenic regions.

#### Locus selection and models

The framework of a BioBin analysis is to determine biological features upon which data will be binned, such as genes, pathways or intergenic regions, and execute bin generation using LOKI. For locus binning, BioBin follows an allele frequency threshold approach using the non-major allele frequency (NMAF). NMAF is defined as 1 minus the frequency of the most common allele, and at biallelic markers, NMAF and minor allele frequency (MAF) are interchangeable. BioBin allows variants below a user-specified NMAF in the case or the control group to be binned, thereby facilitating the aggregation of both potential risk and protective variants. In order to alleviate increased Type I error, BioBin also gives an option to use the minimum of the NMAF in either case or control group as the value to test against the given NMAF threshold [11].

BioBin provides multiple disease model options for determining individual contribution in a bin. This includes additive, dominant, or recessive encoding allowing the user to test specific hypotheses using these inheritance patterns. The default option utilizes additive encoding, where each allele adds to an individual bin score.

#### Customization

The power of BioBin becomes apparent in the flexibility provided to the user, which makes the software applicable in a number of low frequency variant analysis pipelines. In addition to the predefined biologically-informed binning strategies, BioBin allows for customized knowledge, adjustable multi-level feature types, filtering strategies and individual variant weighting.

LOKI contains diverse knowledge from many databases, which together provide variant details, region annotations, and group relationships. To accommodate a wide variety of analyses, the user can choose to include or exclude any source in LOKI. Additionally a user can expand on the predefined knowledge contained within this biorepository as LOKI specification and code are open source allowing the addition of desired database sources. For instance, users may specify additional knowledge through the use of plain text files that can define regions, group or variant weights, and roles. Examples of these input files are provided in the BioBin manual (https://ritchielab.com/software/biobin-download). As part of the customization available, BioBin also accepts custom role files, which contain single variant or region annotations. This file can be used to exclude or specifically include variants based on the results returned from an annotation tool such as Polyphen, SIFT, or SNPEff [24–26].

#### Variant weighting

To adjust statistical power in a rare variant analysis, BioBin provides the option of weighting loci according to the weighted sum statistic proposed by Madsen and Browning [27], in which the weight of a variant is inversely proportional to its MAF. Multiple weighting schemes are provided which use different populations to calculate these locus weights. For instance, in *control* weighting, weighting is calculated based only on the control population. This weighting represents an exact implementation of Madsen and Browning weighting [27]. Because determining allele rarity solely on the control population has been shown to potentially inflate type I error [28, 29], BioBin implements other weight models allowing the user a means by which to utilize variant weighting while controlling this error. In the *maximum* model, the weight is the maximum calculated for the case and control populations, while the *minimum* model uses the minimum weight in these populations. *Overall* weighting calculates the weight using the entire overall population, regardless of case or control status. The *overall* weighting scheme is nearly equivalent to the Madsen and Browning weighting implementation in SKAT [30, 31]. These methods will be equivalent in the circumstance where there are no cases, or there is completely missing case or control population for a given locus. Finally, BioBin can also incorporate custom weights based on the user’s prior knowledge.

### Simulations

Simulation testing was performed to evaluate type I error and power with the various weighting schemes within BioBin (control only, maximum, minimum, overall, and no weighting) using two standard statistical tests: logistic regression and the Wilcoxon rank sum test. SeqSIMLA2 [32] a tool commonly used to simulate unrelated case control sequence data for various genetic analyses, was used to generate all sequence files, which then served as input for BioBin in the present analyses. To generate a reference sequence for SeqSIMLA2, the 1000 Genomes Project Phase I VCF file was parsed to obtain allele frequencies for all gene regions in the autosomes specific to individuals of European descent. A customized python script was then used to randomly select sites from this allele frequency file and generate a reference sequence in the format required by SeqSIMLA2, a binary zip file with each row being a sequence and each column a site. This custom script can be found in Additional file 1. For each SeqSIMLA2 dataset simulation, a sequence file of 10,000 reference samples was created with the number of generated markers varying in relation to the biologically-based bin size being tested (specified under *Type I Error Analysis and Power Analysis*). SeqSIMLA2 was then run with simulation parameters specific to the analysis being performed (see Table 1 for parameters). The output plink files were converted to VCF, and a BioBin variant binning analysis was performed with a MAF cutoff of 5 %. This was followed by statistical analysis using logistic regression and the Wilcoxon rank sum test. Type I error and power were evaluated for each weighting method.

**Table 1:** Simulation parameters. Parameters for the type I error analysis and the power analysis simulations performed using SeqSIMLA2

Testing parameter	Type I error analysis	Power analysis
Bin size assessed	*Gene-sized bin:* 25 kb (50 ± 10 variants) *XL_Gene sized bin:* 100 kb (200 ± 10 variants)	*Gene-sized bin:* 25 kb (50 ± 10 variants)
	*Pathway sized bin:* 2–50 gene-sized bins (100–2500 ± 10 variants)	
Number of simulations	1000	1000
Sample size	500 cases, 500 controls	500 cases, 500 controls
Disease prevalence	5 %	5 %
Number of causal variants	N/A	10
Odds ratio (OR)	1	1.25, 1.5,1.75, 2, 2.5, 3, 4, 5
Variant weighting	No weighting	No weighting
	Control only weighting	Control only weighting
	Minimum weighting	Minimum weighting
	Maximum weighting	Maximum weighting
	Overall weighting	Overall weighting
Statistical test	Logistic regression	Logistic regression
	Wilcoxon	Wilcoxon

#### Type I error analysis

Parameters for the type I error simulation analysis are listed in the left pane of Table 1. Type I error was assessed by performing three different tests, each varying in the size of the biological bin, as we attempted to simulate datasets that roughly correspond to gene-level and pathway level analyses. The choice of size for gene-based simulations is largely debated, and we decided to test three different bin sizes to accommodate various binning analyses, and to explore the relationship between bin size and type I error. These tests include a 25 kb gene-sized bin (referred to as average gene) composed of 50 variants (standard deviation = 5), a large 100 kb gene-sized bin (referred to as XL gene throughout this work) composed of 200 variants (standard deviation = 5), and a pathway bin composed of 2–50 gene-sized bins, or 100–2500 variants (standard deviation = 5). We chose 50 variants to represent an average sized gene bin by consulting the autosomal variant site statistics reported by 1000 Genomes Project [14, 33] and calculating a rough estimate for the number of possible variants expected in 25 kb, an approximation for median gene size [34]. For each simulation, the specific number of variants was randomly determined. For example, each pathway dataset simulation could contain anywhere from 100 to 2500 variants. Type I error was estimated with 1000 null dataset simulations for each bin size using an odds ratio (OR) of 1, and assessing significance with an α of 0.05 for both regression and Wilcoxon.

#### Power analysis

To assess the statistical power of each weighting method, power analyses were performed with 1000 simulations of an average sized 25 kb gene bin, containing 50 variants (standard deviation = 5), as described in the right pane of Table 1. For each simulation, 10 causal variants or disease sites were randomly selected in the binned locus. Eight independent simulation tests were performed for each weighting scheme in which the OR of the causal variants was varied as 1.25, 1.5, 1.75, 2, 2.5, 3, 4, and 5. Power was assessed for each of these OR analyses with logistic regression and Wilcoxon using a significance criteria of 0.05.

## Results and discussion

BioBin is an innovative variant collapsing method that provides a flexible infrastructure for biologically informed variant binning adaptive to individual user needs. In this work, we evaluated four weighting schemes provided within BioBin: control, minimum, maximum and overall weighting, in addition to the no locus weighting option. These weighting methods were examined using two standard burden tests: regression and the Wilcoxon rank sum. While multiple studies have performed exhaustive comparisons of statistical tests for rare variant analyses [35–37], the focus of BioBin is to build versatile and biologically relevant bins rather than to implement a particular statistical analysis. BioBin can provide the necessary files for a user to implement his or her statistical test of choice; this provides the user with freedom to choose the statistical test that is most appropriate for his/her hypothesis. We chose to specifically focus on regression and the Wilcoxon rank sum test as these are very commonly used methods in rare variant analyses [27, 38–41].

### Type I error analysis

**Fig. 5:**
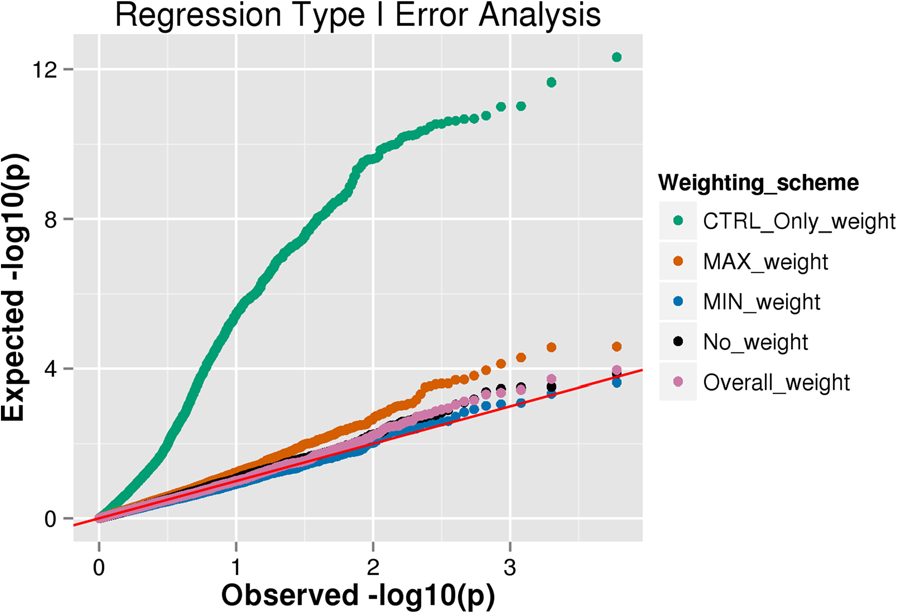
Logistic regression type I error analysis. A comprehensive quantile-quantile plot for the type I error logistic regression analysis showing the combined p-value distribution from the average gene, XL gene, and pathway simulations. The different colors are representative of the various weighting schemes in BioBin that were analyzed

**Fig. 6:**
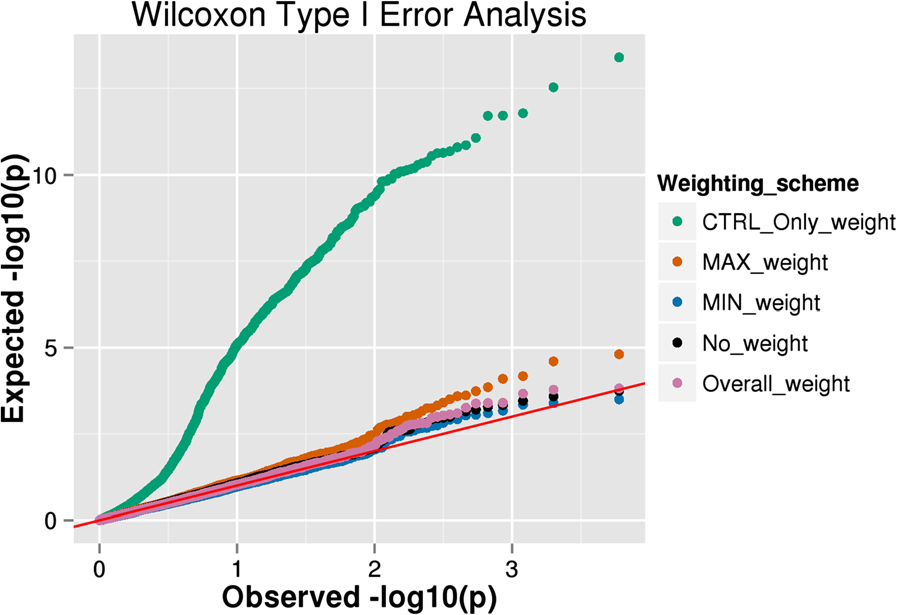
Wilcoxon type I error analysis. A comprehensive quantile-quantile plot for the type I error Wilcoxon rank sum analysis showing the combined p-value distribution from the average gene, XL gene, and pathway simulations. The different colors are representative of the various weighting schemes in BioBin that were analyzed

Results of the type I error analysis using logistic regression and Wilcoxon for bins of all biological-based sizes are presented as quantile-quantile (QQ) plots in Figs. 5 and 6, respectively. Both figures are comprehensive plots combining simulation p-value results from the average gene, XL gene and pathway analyses. The simulation results indicate that weighting using only the control population (*CTRL_ONLY_weight*) drastically inflates the type I error in both tests examined. Similar to the observations made by Lemire [28] and Pearson [29] in which allele rarity based solely on the control population introduces a bias, we also observe that weight calculations using only this phenotypic class increase type I error. A variant selection bias is created since there is an upper limit for the frequency of variants in the controls, but there is no bound for variant frequencies in the case population. This error becomes even more inflated when the size of the bin is increased from that of an average gene to a pathway, as evident in the additional material, thus introducing a spurious correlation that can confound results. BioBin implements other weight models where frequency thresholds are established using cases and controls, thereby imposing an upper frequency bound in both phenotypic classes, providing the user a means by which to utilize variant weighting while controlling type I error. Of the weighting models tested, minimum weighting (*MIN_weight*) for the case and control populations presented the lowest type I error rate. The weights calculated from the overall allele frequency (*Overall_weight*), which is a common implementation of the Madsen and Browning test in current online tools, are mostly well controlled. Maximum weighting (*MAX_weight*) had a greater type I error rate than overall, minimum, and no weighting, but still a lower false positive rate when compared with control only estimates, especially when the bin size was increased.

### No correlation between significance and bin size (except with control weighting)

Table 2 displays the type I error results for the different weighting schemes and different bin sizes. The majority of the weighting methods have a type I error controlled around 5 %, with the exception of maximum weighting which is closer to 8–9 % and control weighting which is dramatically higher. As seen in Figs. 5 and 6, control weighting yields a greatly inflated type I error, and further, Table 2 suggests that the amount of error is highly correlated with the specific bin structure. Further evidence of this can be seen in the supplemental section, where we show the QQ plots for each specific biological structure independently. In order to assess the role that the bin size plays in the false positive rate, we ran a logistic regression using the p-value of a simulated bin as the outcome and number of loci in a bin as the predictor. We chose logistic regression in this case because the outcome (p-value) is bounded between 0 and 1, but similar trends are seen using probit regression and ordinary least squares regressions as well (data not shown). The results, shown in Table 3, clearly indicate that for control weighting, an increase in the number of variants in a bin drastically increases the chance of a false positive finding. All other variant weighting strategies did not show any significant relationship between bin size and false positive rate. As discussed above, this trend is explained by the bias imposed when weighting variants using allele frequency thresholds calculated only from the control population, as no upper bound is imposed for case variant frequencies. However, when we impose bounds by weighting via the maximum, minimum or overall methods, we see a lower type I error rate.

**Table 2:** Type I error results. The Type I error simulation results displayed per BioBin weighting scheme tested, biological bin size assessed, and statistical analysis test

Statistical test	Bin size	Weighting scheme				
		Control only weight	Max weight	Min weight	No weight	Overall weight
Logistic	Gene	0.106	0.079	0.042	0.060	0.052
	XL Gene	0.343	0.096	0.042	0.062	0.057
	Pathway	0.847	0.090	0.040	0.066	0.059
Wilcoxon	Gene	0.064	0.061	0.052	0.060	0.057
	XL Gene	0.153	0.068	0.043	0.056	0.055
	Pathway	0.795	0.085	0.045	0.072	0.056

**Table 3:** Correlation of bin size and significance. Using the control weighting, the larger bins result in a higher chance of a false positive finding, showing a correlation between bin size and p-value. All other weighting strategies have false positive rates independent of bin size

	Logistic		Wilcoxon	
	Beta (SE)	*p*-value	Beta (SE)	*p*-value
Control	−6.83e-3 (6.13e-4)	7.51e-29	−5.77e-3 (4.42e-4)	6.07e-39
Max	4.08e-5 (8.40e-5)	0.627	−4.75e-5 (8.39e-5)	0.572
Min	2.88e-5 (8.39e-5)	0.732	5.06e-5 (8.39e-5)	0.546
None	−2.43e-5 (8.38e-5)	0.772	−3.15e-5 (8.38e-5)	0.707
Overall	3.64e-5 (8.38e-5)	0.664	2.05e-5 (8.38e-5)	0.806

### Power analysis

**Fig. 7:**
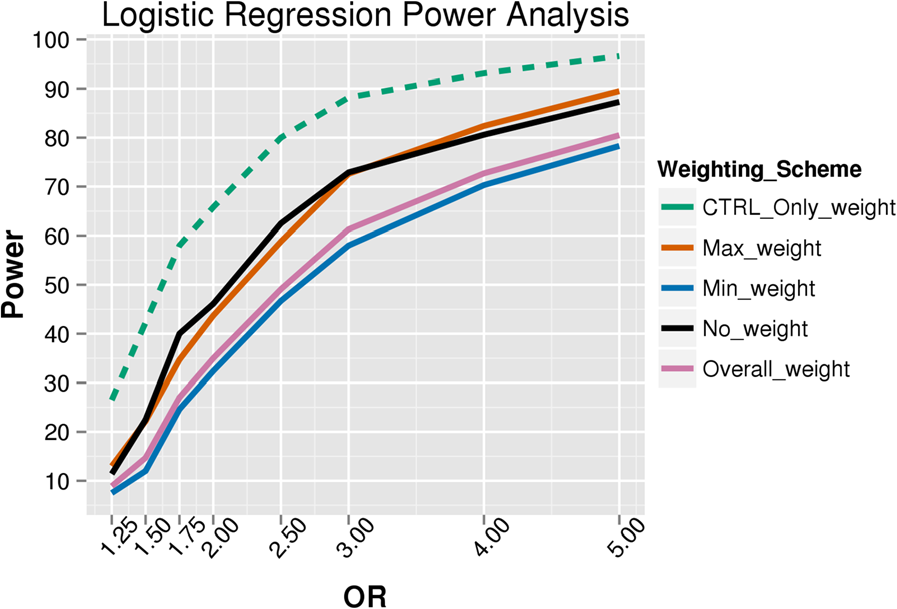
Logistic regression power analysis. Statistical power estimates for multiple weighting methods assessed at varying odds ratios using logistic regression analysis. Results are based on 25 kb bins containing 50 (±10) variants with 10 of these representing causal variants. The different colors represent the various weighting schemes in BioBin that were analyzed. Control only weighting is presented with a dashed line to draw attention to the inflated type I error (presented in Fig. 6 and Additional files 2: Figure S1 and Additional file 3: Figure S2) of this weighting method

**Fig. 8:**
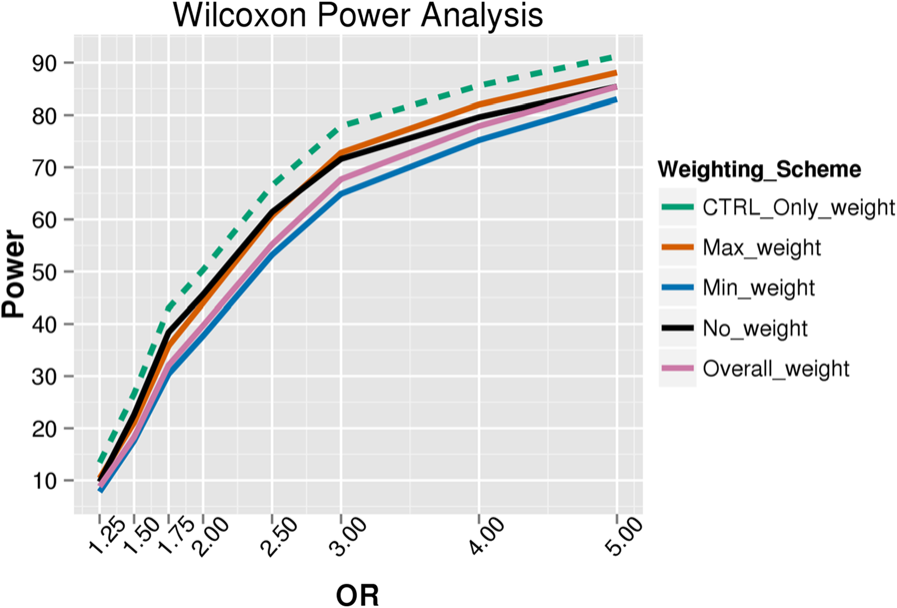
Wilcoxon power analysis. Statistical power estimates for multiple weighting methods assessed at varying odds ratios using the Wilcoxon rank sum test. Results are based on 25 kb bins containing 50 (±10) variants with 10 of these representing causal variants. The different colors represent the various weighting schemes analyzed in BioBin. Control only weighting is presented with a dashed line to draw attention to the inflated type I error (presented in Fig. 5 and Additional file 2: Figure S1) of this weighting method

The power analysis simulation results are shown in Figs. 7 and 8 for the logistic regression and Wilcoxon rank sum analysis, respectively. The most powerful BioBin weighting method was using the control weighting (*CTRL_ONLY_weight*). However, this weighting scheme has an inflated false positive rate, which is further magnified when the bin size is increased, as seen in Figs. 5 and 6 and Additional file 2: Figure S1. The most powerful BioBin weight with a more controlled type I error is the maximum weight, which has greater power with a Wilcoxon test than with logistic regression (however, this strategy has the second highest type I error rate around 8–9 %). To further evaluate sensitivity in the context of bin size for the minimum, maximum and overall weighting methods, we performed additional power analyses with 1000 simulations of a 100 kb sized gene bin, containing 200 total variants (standard deviation = 5) with 10 of these being causal or disease sites, and varying the OR of the causal variants from 1.25 to 5 (results not shown). Power of the 100 kb (200 variant) gene bins was assessed using logistic regression with a significance criteria of 0.05 and compared with that of the average 25 kb (50 variant) gene bins. Results of this comparison show consistently decreased power in the larger gene bins likely due to noise introduced by the addition of neutral variants, while maintaining the same number of causal variants as in the 25 kb bins. While future work will aim at testing these observations by evaluating varied proportions of casual to neutral variants, early indications implicate this ratio of variants as the primary driver in sensitivity for a constant effect size (OR).

In the present simulations, the no loci weighting option in BioBin presents as statistically more powerful than both overall and minimum weighting. We believe this to be a result of the specific simulation parameters chosen for this analysis, and would likely be altered by the number of binned loci, the allele frequencies of the variants, the direction of the variant effect, and the sample size. Additional simulations were performed in an attempt to demonstrate the influence of chosen parameters on our simulation analyses. We performed comparable power analyses to those noted above, but restricted the selection of variants to only those having a MAF below 5 %, thereby causing all selected disease sites to be binned, and increased the number of casual variants to 20 (standard deviation = 5). The results of this analysis show that simulations without loci weighting had the lowest power across all tested ORs (1.25, 1.75, 2.5, 4 and 5) when compared with all other weighting methods. These results suggest that weighting approaches may have a larger influence on power when the selected disease sites are rare since different results were observed when disease sites with probabilities inversely proportional to the MAF are chosen. Overall, the power results are heavily influenced by simulation methodology, and future work will aim at performing a thorough sweep of simulation parameters and weighting methods in BioBin.

### Future work

We have performed a preliminary study on incorporating select burden and dispersion-based statistical tests as well as multiple phenotype analysis capabilities into the framework of BioBin [12]. Future work will include comprehensive testing of burden and dispersion methods as well as dissemination of an updated BioBin software package, BioBin 2.2.0, with these additional features.

## Conclusions

Overall, BioBin is a powerful and versatile tool for the knowledge-guided biological binning and analysis of low frequency variants in sequence data. BioBin uses a diverse repository of data from a multitude of public sources, and thereby circumvents the necessity of manually curating biologically important data for variant collapsing. BioBin provides users with a flexible and customizable framework to analyze sequence data and uncover novel associations with complex traits.

## Additional files

Additional file 1: Script for generating reference sequence. Python script used to generate a reference sequence file for input into SeqSIMLA2 simulation software. The allele frequency file used in the script was obtained by parsing the protein coding regions of the autosomes in the 1000 Genomes Project VCF file. Additional specifications include the number of reference samples to generate and the number of markers to include in the reference file. (DOCX 14 kb) Additional file 2: Figure S1. Logistic regression type I error per biological feature. QQ plots for the type I error logistic regression analysis showing the p-value distribution from the average gene (**a**), XL gene (**b**), and pathway (**c**) simulations. The different colors represent various BioBin weighting schemes analyzed. (PNG 170 kb) Additional file 3: Figure S2. Wilcoxon type I error per biological feature. QQ plots for the type I error Wilcoxon Rank Sum analysis showing the *p*-value distribution from the average gene (**a**), XL gene (**b**), and pathway (**c**) simulations. The different colors represent various BioBin weighting schemes analyzed. (PNG 147 kb)
