# Supplementary material for: A biologically informed method for detecting rare variant associations
Source: BioData Min. 2016 Aug 30;9(1):27. doi: 10.1186/s13040-016-0107-3 (PMC5006419; doi:10.1186/s13040-016-0107-3)
Supplement: Additional file 1: — Script for generating reference sequence. Python script used to generate a reference sequence file for input into SeqSIMLA2 simulation software. The allele frequency file used in the script was obtained by parsing the protein coding regions of the autosomes in the 1000 Genomes Project VCF file. Additional specifications include the number of reference samples to generate and the number of markers to include in the reference file. (DOCX 14 kb) [file 13040_2016_107_MOESM1_ESM.docx]

#!/usr/bin/env python

import argparse

import sys

import struct

import random

def iter_sample_fast(iterable, samplesize):

results = []

iterator = iter(iterable)

# Fill in the first samplesize elements:

for _ in xrange(samplesize):

results.append(iterator.next())

random.shuffle(results) # Randomize their positions

for i, v in enumerate(iterator, samplesize):

r = random.randint(0, i)

if r < samplesize:

results[r] = v # at a decreasing rate, replace random items

if len(results) < samplesize:

raise ValueError("Sample larger than population.")

return results

if __name__ == "__main__":

# Add our command line arguments

parser=argparse.ArgumentParser(description="Generate SeqSIMLA file from allele frequencies")

parser.add_argument("--file", "-f", help="Allele frequency file (one MAF per line)", required=True, type=argparse.FileType('r'))

parser.add_argument("--samples", "-s", help="Number of reference samples to generate", type=int, required=True)

parser.add_argument("--markers", "-m", help="Number of markers to include in reference file", type=int, required=True)

parser.add_argument("--output", "-o", help="SeqSIMLA output file", required=True)

args = parser.parse_args();

# read the file of MAFs, and take a random sampling of # of markers

maf_list = iter_sample_fast((float(l) for l in args.file), args.markers)

# open the seqsimla output file

out_f = file(args.output, 'wb')

# write the header

out_f.write(struct.pack('>II', args.markers, args.samples))

# now, for each sample

for s in xrange(args.samples):

b = 0

# For each marker

for i in xrange(args.markers):

# generate a random number, and if it

b = b | ((random.random() < maf_list[i]) << 7 - (i % 8))

if i % 8 == 7:

out_f.write(struct.pack('B',b))

b = 0

if len(maf_list) % 8 != 0:

out_f.write(struct.pack('B',b))

out_f.close()
