# Supplementary figures and images for: A biologically informed method for detecting rare variant associations
Source: BioData Min. 2016 Aug 30;9(1):27. doi: 10.1186/s13040-016-0107-3 (PMC5006419; doi:10.1186/s13040-016-0107-3)

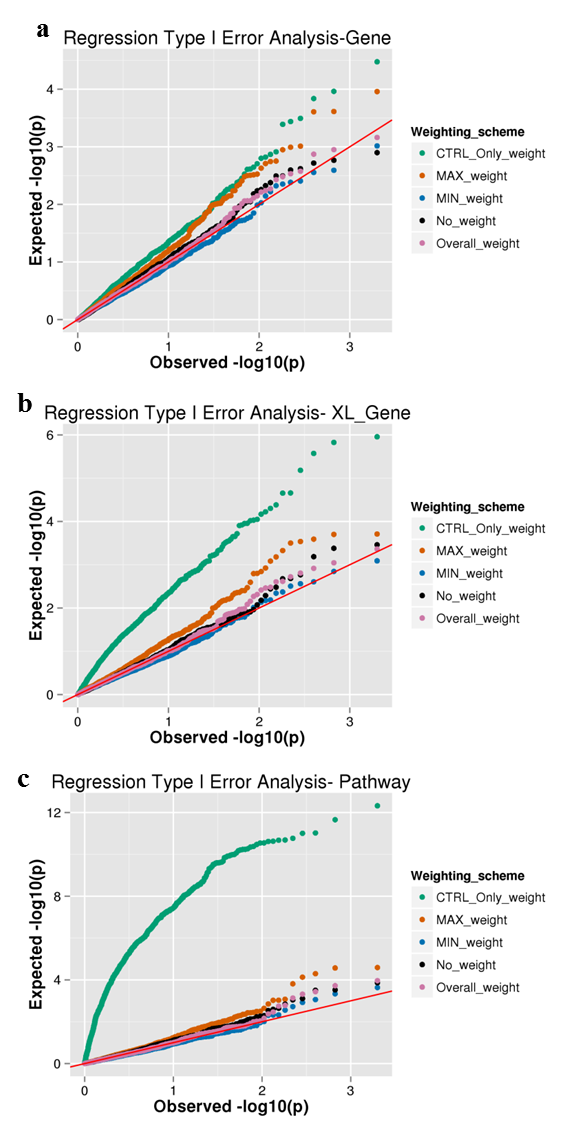

Supplement: Additional file 2: Figure S1. — Logistic regression type I error per biological feature. QQ plots for the type I error logistic regression analysis showing the p-value distribution from the average gene (a), XL gene (b), and pathway (c) simulations. The different colors represent various BioBin weighting schemes analyzed. (PNG 170 kb) [file 13040_2016_107_MOESM2_ESM.png]

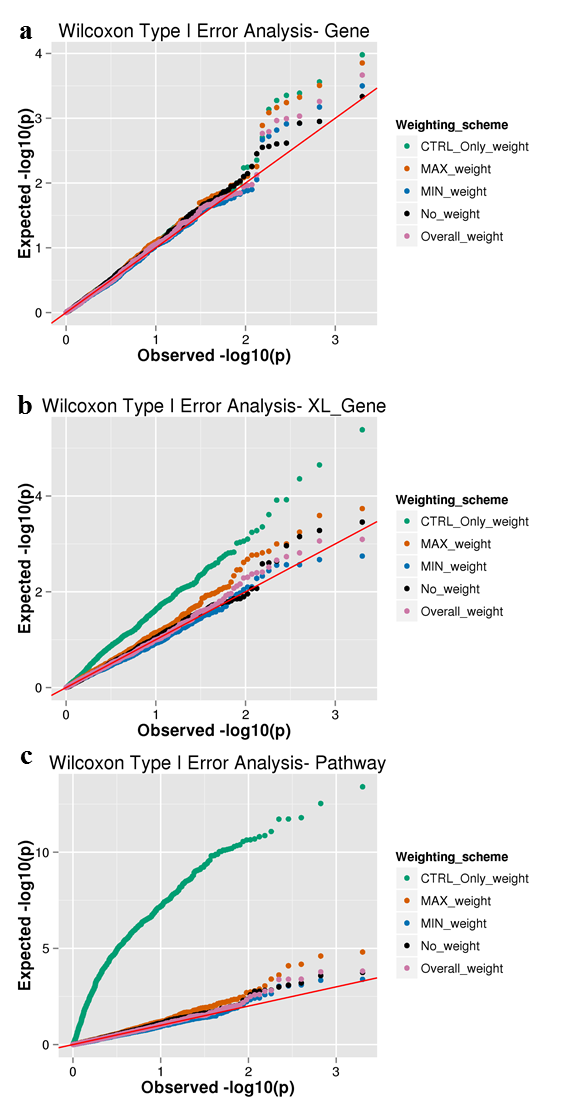

Supplement: Additional file 3: Figure S2. — Wilcoxon type I error per biological feature. QQ plots for the type I error Wilcoxon Rank Sum analysis showing the p-value distribution from the average gene (a), XL gene (b), and pathway (c) simulations. The different colors represent various BioBin weighting schemes analyzed. (PNG 147 kb) [file 13040_2016_107_MOESM3_ESM.png]
